# Evaluating the efficacy of a novel home-based oral food challenge protocol for pediatric food protein induced enterocolitis syndrome

**DOI:** 10.1186/s13223-025-00994-z

**Published:** 2025-10-30

**Authors:** Emily G. Morris, Peter W. Huan, Jennifer L. P. Protudjer, Lucy Li, Natalie Rondilla, Jeevan Abraham, Harold Kim

**Affiliations:** 1 Department of Medicine, Western University, London, ON Canada; 2 Department of Pediatrics and Child Health, Rady Faculty of Health Sciences, Max Rady College of Medicine, University of Manitoba, Winnipeg, MB Canada; 3 Children’s Hospital Research Institute of Manitoba, Winnipeg, MB Canada; 4 Department of Food and Human Nutritional Sciences, Faculty of Agricultural and Food Sciences, University of Manitoba, Winnipeg, MB Canada; 5 Institute of Environmental Medicine, Karolinska Institutet, Stockholm, Sweden; 6 Division of Clinical Immunology and Allergy, Department of Paediatrics, University of Toronto, Toronto, ON Canada; 7 Western University, London, ON Canada; 8 Wilfrid Laurier University, Waterloo, ON Canada; 9 Division Clinical Immunology and Allergy, Department of Medicine, Western University, London, ON Canada; 10 Faculty of Health Sciences, McMaster University, Hamilton, ON Canada

**Keywords:** Food protein–induced enterocolitis syndrome, Oral food challenges, Pediatrics, Food allergies

## Abstract

**Background:**

Oral food challenges (OFCs) are considered the gold standard for diagnosis of food protein–induced enterocolitis syndrome (FPIES), a non-immunoglobulin E mediated gastrointestinal food allergy characterized by delayed, repetitive vomiting, lethargy, and sometimes diarrhea, primarily affecting infants and young children. Our modified approach to OFCs involves smaller, gradually increased doses to mitigate the risk of severe reactions. We aimed to measure the successful completion of this OFC protocol.

**Methods:**

In a retrospective chart review, patients age < 18 years, who had 1 + episode of acute FPIES between 2015 and 2023 were identified using an allergy clinic database. Patients underwent OFCs with home up dosing every 2–4 weeks. Steps included 1%, 2%, 5%, 10%, 20%, 30%, 40%, 60%, 80%, and 100% of the final serving amount. The primary outcome was successful completion, i.e. absence of severe reactions during the OFC protocol and 1 year after. Data were analysed using logistic regression and reported as odds ratios (OR) and 95% confidence intervals (95% CI). Results were adjusted for multiple allergic comorbidities, age of FPIES onset, and biological sex.

**Results:**

Among 47 patients who began the OFC protocol, 38 (80.85%) completed it without significant reactions. Of the 9 (19.14%) who did not complete the protocol, 4 (44.4%) paused due to reactions, and 5 (55.6%) paused due to non-FPIES symptoms. The 4 reactors paused due to mild-to-moderate reactions; there were no severe reactions during the protocol. There were no significant associations identified between OFC completion and severity of symptoms (OR 1.05; 95% CI 0.24–4.71; *p* = 0.94); age at onset of symptoms (OR 0.99; 95% CI 0.94–1.02; *p* = 0.58); or age of starting OFC (OR 1.00; 95% CI 0.98–1.02; *p* = 0.90). Patients who reacted to milk tended to be less likely to complete the protocol than those reacting to other foods (OR 0.28; 95% CI 0.07–1.06; *p* = 0.06).

**Conclusions:**

This study supports the potential for a home-based gradual approach to OFCs in FPIES, evidenced by a high completion rate and no severe reactions.

## Background

Food protein–induced enterocolitis syndrome (FPIES) is a non-immunoglobulin E (IgE)-mediated food allergy, predominantly affecting infants [1]. FPIES is characterized by reactions including vomiting, diarrhea, lethargy, pallor, abdominal distention, and in more severe cases, hypotension and shock [2]. Common triggers are soy, cow’s milk, and grains, with symptoms typically emerging within 1-to-4 h of ingestion [1, 3]. A chronic form of FPIES is also noted, where ongoing exposure to trigger foods leads to persistent symptoms and failure to thrive. The diagnosis is mainly clinical, based on a history of repeated reactions to the same food triggers and improvement upon their removal from the diet [4]​. Although most patients have outgrown their FPIES reaction in the school-age years, it has also been recognized to persist into adolescence or even present for the first time in adulthood, underscoring the variability in its clinical presentation [5]. 

The natural history of FPIES suggests a generally favorable prognosis, with most affected infants experiencing spontaneous resolution within the first few years of life. Approximately 60–90% of affected children achieve tolerance the triggering food by 3 to 5 years of age, although the rate varies depending on the food trigger involved [6–8]. Milk and soy are the predominant food triggers across multiple study populations, whereas secondary trigger foods differ among populations based on cultural dietary practices—for example, fish is introduced earlier in the Italian diet compared to North American populations [7–10]. Observed differences in cross reactivity between different FPIES triggers between populations were also hypothesized to due to the order of food introduction and potential microbiome differences [8, 9]. Infants whose FPIES resolves tend to have excellent outcomes, with normal growth, development, and nutritional status, provided appropriate dietary management was implemented during the active phase of the disease [11]. 

Oral food challenges (OFCs) are considered the gold standard for confirming FPIES [12]. Traditional OFCs involve administering a food protein dose of 0.06–0.6 g/kg in three equal doses over a 30-to-60-min period. If no symptoms occur within 2-to-3 h, a full age-appropriate food serving is given, followed by another 4-h monitoring period [2]. However, this approach has been criticized for the risk of severe reactions, including hypotension and shock, especially since symptoms can manifest or escalate hours after ingestion [13, 14].​

To address these concerns, we developed a modified OFC protocol with smaller, gradually increasing doses every 2 to 4 weeks over the course of 10-months. Patients who underwent this OFC protocol completed up dosing at home, with the following incremental percentages of a final serving amount: 1%, 2%, 5%, 10%, 20%, 30%, 40%, 60%, 80%, and 100% of an appropriate serving amount. This new method aims to reduce the risk of severe reactions and allows for monitoring of delayed or chronic FPIES.

Several clinical and demographic factors may influence outcomes in patients undergoing OFC for FPIEs. Previous studies suggest that a history of atopic dermatitis, asthma, allergic rhinitis, and family history of atopy may be associated with increased susceptibility to food allergies and could potentially influence the severity of FPIES [2, 3, 15]. Additionally, variables such as gestational age, sex, and weight at time of first symptom onset have been examined in prior research as potential risk modifiers of allergic disease severity or resolution [10, 12, 16]. Understanding of these factors is critical in identifying children at higher risk for persistent FPIES symptoms and guide individualized clinical management. Considering the previously mentioned demographic and cultural variations in food introduction practices and their impact on FPIES triggers, the specific food allergens investigated in this study were selected based on priority allergens identified in Canadian populations, including cow’s milk, egg, peanut, tree nuts, fish, shellfish, soy, wheat and triticale, mustard, and sesame [16]. 

This study aims to evaluate the efficacy and safety of our modified OFC protocol specifically tailored to FPIES, in the context of these influential factors. Identifying demographic and clinical predictors of OFC outcomes may ultimately refine FPIES management, improve patient safety, and enhance our ability to predict long-term tolerance in affected children.

## Methods

### Patient selection

This retrospective cohort study included 47 participants ages 5 months to 13 years old from an allergy outpatient clinic in Kitchener-Waterloo, Ontario, Canada. Informed consent was obtained from parents/guardians for the use of medical records in research. Patients selected were those who had a diagnosis of FPIES between January 2015 and April 2024, defined as meeting one major criterion and at least three of the minor criteria determined by the AAAAI 2017 guidelines [2]. The Telus Health electronic medical record systems at the outpatient allergy clinic was used to collect the relevant de-identified patient data.

Inclusion criteria was defined as pediatric patients of the outpatient clinic, who had at least 1 episode of acute FPIES whether mild-to-moderate or severe, defined in accordance with AAAAI 2017 FPIES guidelines [2]. (reference the website hyperlink—Table 1 of guideline).

**Table 1:** Study population participants characteristics (*N* = 47)

Characteristic	Study population (*N* = 47)	
	*n*	%
Male	27	57.4%
Other allergic comorbidities ±		
Atopic dermatitis	21	44.6%
Asthma	10	21.3%
Allergic rhinitis	6	12.8%
IgE mediated food allergy diagnosis	15	31.9%
Other prescription medications during OFC*	19	40.4%
Family history of atopy	29	61.7%

	*n*	Mean ± SD
Age of first FPIES reaction in months	46	12.50 ± 19.7
Age of starting modified FPIES OFC in months	42	40.5 ± 34.2
Gestational age in weeks	18	38.3 ± 2.6

*Other prescriptions noted included antihistamines, short acting beta agonists, long acting muscarinic antagonists, inhaled corticosteroids, epinephrine autoinjectors, proton pump inhibitors, and systemic corticosteroids

± Not mutually exclusive co morbidities

*IgE* immunoglobulin E, *OFC* oral food challenges, *FPIES* food protein induced enterocolitis

Exclusion criteria were defined as patients demonstrating reactions without satisfaction of acute FPIES symptoms, for example, isolated IgE reactions without vomiting. Additionally, those with diagnostic uncertainty, such as removal of culprit food did not result in symptom resolution, despite meeting other characteristics of FPIES.

### Data abstraction

From the clinic database, we extracted information pertinent to consider as covariates for risk factors for FPIES, such as history of atopic dermatitis, asthma, and allergic rhinitis. Additionally, co-variants information such as IgE mediated reactions to food allergens, and food protein–induced allergic proctocolitis (FPIAP) were collected. We also collected demographic data such as sex, gestational age, weight at FPIES onset, weight at start of OFC protocol, other medical conditions, and family history of atopy. This information was gathered primarily through parent-reported history during initial clinical consultations and subsequent follow-up appointments, supplemented by clinical examination findings where available. Furthermore, we collected information on the completion of OFC protocol and specifics of any reaction during the OFC protocol. Reactions during the OFC protocol were stratified as either mild-moderate or severe acute FPIES according to Table II in the AAAAI 2017 FPIES guidelines [2]. By examining these factors, our goal was to refine understanding of effective FPIES management strategies and to enhance patient outcomes through safer OFC protocols.

### The protocol

**Fig. 1:**
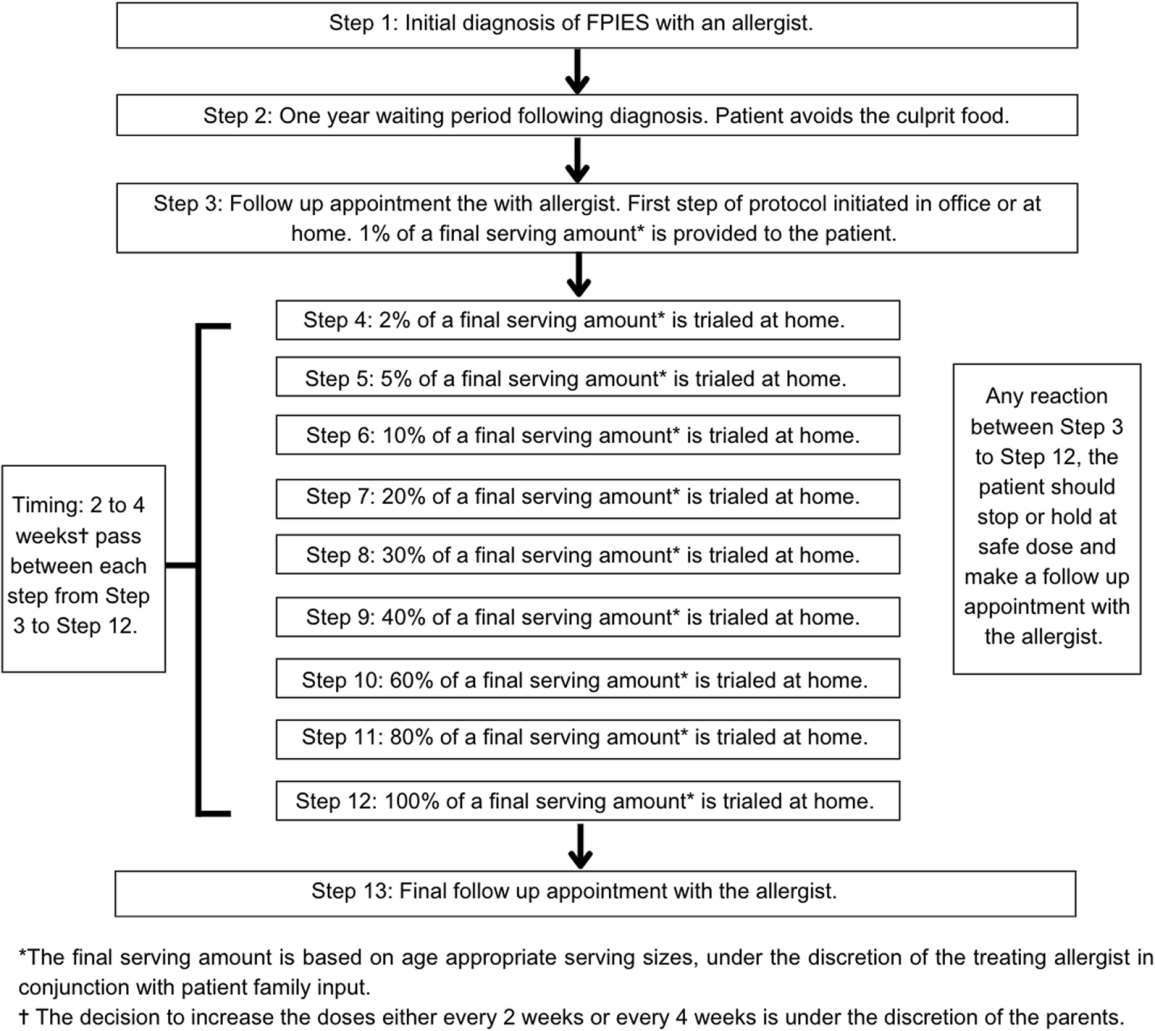
Modified FPIES OFC protocol. Abbreviations: oral food challenges (OFC), food protein induced enterocolitis (FPIES)

Figure 1 illustrates a schematic diagram of the revised FPIES OFC protocol. The revised protocol for FPIES OFC is implemented 1 year after initial diagnosis of FPIES with the specialist allergist physician. There is no defined age threshold for initiating this protocol. The OFC involves gradually increasing doses of the culprit food every 2 to 4 weeks over the course of up to 10-months. Each up-dosing step and maintenance dosing occurs at home, after consultation and education with an allergist. The following incremental percentages of a final serving amount are used as the steps for each up dose: 1%, 2%, 5%, 10%, 20%, 30%, 40%, 60%, 80%, and 100% of a final serving amount. The decision to increase the doses either every 2 weeks or every 4 weeks is under the discretion of the parents. The final serving amount is based on age-appropriate serving sizes, under the discretion of the treating allergist in conjunction with patient family input. During the protocol, there is no specific follow-up scheduled. Patients are to make a follow up appointment should there be a reaction. Following this protocol, patients are seen in follow up by the allergist.

### Study outcomes

Our primary outcome was successful completion of our modified OFC protocol, operationalized as the ability to complete the OFC in the absence of severe reactions, which broadly aligns with the criteria outlined in the American Academy of Allergy, Asthma & Immunology (AAAAI) 2017 guidelines [2]. Long-term outcomes and tolerability were further characterized by assessing whether patients successfully completed the protocol and experienced no reactions for at least 1 year afterward. Secondary outcomes included identifying reasons for unsuccessful completion of the protocol, distinguishing between FPIES-related and non-FPIES-related causes, an evaluating the severity of acute FPIES reactions occurring during the OFC;

### Statistical analysis

Data was described (n/N, % mean, median, standard deviation, etc.) to understand candidate variables including patient age, biological sex, time of first reaction, trigger foods/cause of reaction, and similar. We examined differences between types of foods or number of trigger foods eliciting FPIES reactions. These candidate variables were chosen based on their demonstrated relevance in existing literature of age-specific challenges associated with OFCs [17]. Likewise, the offending food agents chosen are based on published literature priority allergens in Canada [16] including cow’s milk, egg, peanut, tree nut, fish, shellfish, soy, wheat and triticale, mustard, sesame and soy.

Comparisons were made using logistic regression (fully adjusted OR, 95% confidence interval, standard deviation) with adjustments made for confounding variables. Multivariable logistic regression was used to adjust for confounders and determine the independent effects of each predictor. In our final model, we adjusted for multiple allergic comorbidities, age of FPIES onset, and biological sex. Tables 4 and 5 include both unadjusted and fully adjusted results, whereas the text details only the adjusted results. Data was analyzed using Stata (version 17, College Station, Texas). Ethical approval for this study was granted by the Hamilton Integrated Research Ethics Board (REB #17339).

## Results

Among the 47 patients who initiated the protocol, 38 (80.85%) completed it without significant reactions. There were 9 patients (19.14%) who did not complete the protocol, 4 patients (8.5%) paused due to reactions, and 5 patients (10.6%) paused due to non-FPIES symptoms.

Our study population was majority male (57.4%; Table 1). Many patients had a family history of atopy (61.7%) and other allergic comorbidities, such as atopic dermatitis (44.6%). Other prescriptions noted included antihistamines, short acting beta agonists, long-acting muscarinic antagonists, inhaled corticosteroids, epinephrine auto injectors, proton pump inhibitors, and systemic corticosteroids.

Table 2 presents the characteristics of the nine patients who did not complete the protocol, and Table 3 shows the clinical characteristics of patients with acute FPIES with positive reactions in the OFC. As noted, all (100.0%) of the non-FPIES reactors had other IgE-mediated food allergy diagnosis, compared to none (0.0%) in the FPIES reactors cohort. Similarly, the non FPIES reactors 100.0% were noted to have other allergic comorbidities such as atopic dermatitis, asthma, and allergic rhinitis, whereas the FPIES reactors only 25.0% endorsed these comorbidities. The majority (60.0%) of non FPIES reactors were noted to have reacted to eggs.

**Table 2:** Population characteristics of protocol non-completers: FPIES type reactors versus non FPIES type reactors

Characteristic	FPIES reactor population (*N* = 4)		Non FPIES reactor population (*N* = 5)	
	*n*	%	*n*	%
Male	2	50.0%	4	80.0%
Other allergic comorbidities: Atopic dermatitis, Asthma, Allergic rhinitis	1	25.0%	5	100.0%
IgE-mediated food allergy diagnosis	0	0.0%	5	100.0%
Other prescription medications during OFC*	1	25.0%	2	40.0%
Family history of atopy	3	75.0%	5	100.0%
FPIES culprit food for OFC: Egg	0	0.0%	3	60.0%
FPIES culprit food for OFC: Milk/Dairy	2	50.0%	1	20.0%
FPIES culprit food for OFC: Oats	1	25.0%	1	20.0%
FPIES culprit food for OFC: Tomato	1	25.0%	0	0.0%

	*n*	Mean ± SD	*n*	Mean ± SD
Age of first FPIES reaction in months	4	11.75 ± 13.4	5	5.5 ± 3.2
Age of starting modified FPIES OFC in months	3	48.0 ± 32.7	4	16.9 ± 11.0
Gestational age in weeks	1	40.4 ± 0	1	36 ± 0

*Other prescriptions noted included antihistamines, short acting beta agonists, long-acting muscarinic antagonists, inhaled corticosteroids, epinephrine autoinjectors proton pump inhibitors, and systemic corticosteroids

*OFC* oral food challenges, *FPIES* food protein induced enterocolitis, *IgE* immunoglobulin E

**Table 3:** Clinical characteristics of subjects who had positive reactions in OFC

Characteristic	Patient number			
	1	2	3	4
Sex	Female	Male	Male	Female
Food	Milk	Oats	Milk	Tomatoes
Age of onset (months)	9	7	1	25
Age undertaking OFC (months)	72	20	84	40
Family history of atopy	Yes	Yes	No	Yes
First reaction of acute FPIES (times)	Diarrhea Vomiting (5)	Vomiting (4)	Vomiting (2)	Diarrhea Vomiting
Time to initial reaction from ingestion (h)	0.75	1.5	0	6
Severity of first reaction	Severe	Severe	Mild	Mild
Treatment of initial reaction	ER visit Ondansetron	Self-resolved at home	Self-resolved at home	Self-resolved at home
Protocol load	1% of final serving amount	1% of final serving amount	1% of final serving amount	5% of final serving amount
OFC reaction	Vomiting	Diarrhea Abdo pain	Diarrhea Abdo pain	Diarrhea
Severity in OFC	Mild	Mild	Mild	Mild
Time to reaction from OFC ingestion (h)	0	0	6	6
Treatment	Self-resolved	Self-resolved	Self-resolved	Self-resolved
Confirmatory diagnosis by OFC	Acute FPIES	Acute FPIES	Acute FPIES	Acute FPIES

*h* hours, *OFC* oral food challenges, *FPIES* food protein induced enterocolitis

The five non FPIES reactors stopped the protocol due to various reasons, not in keeping with acute FPIES reactions. Two of the five patients developed atypical FPIES. Additionally, two patients developed mild rashes. One rash developed in conjunction with amoxicillin use, and therefore the patient is planned for reintroduction of our FPIES OFC protocol in the future when no longer on antibiotics. The second patient’s rash occurred at the first dose of the protocol, however after resolution of the rash the OFC was re attempted. Results of the second attempted OFC does not coincide with the timeline of this study and is therefore not reported. Lastly, the final non FPIES reactor developed symptoms of gagging, not consistent with an acute FPIES reaction, but chose not to continue further with the OFC. The decision to stop the OFC despite non FPIES symptoms was decided based on treating physician and parent/patient shared decision-making.

**Fig. 2:**
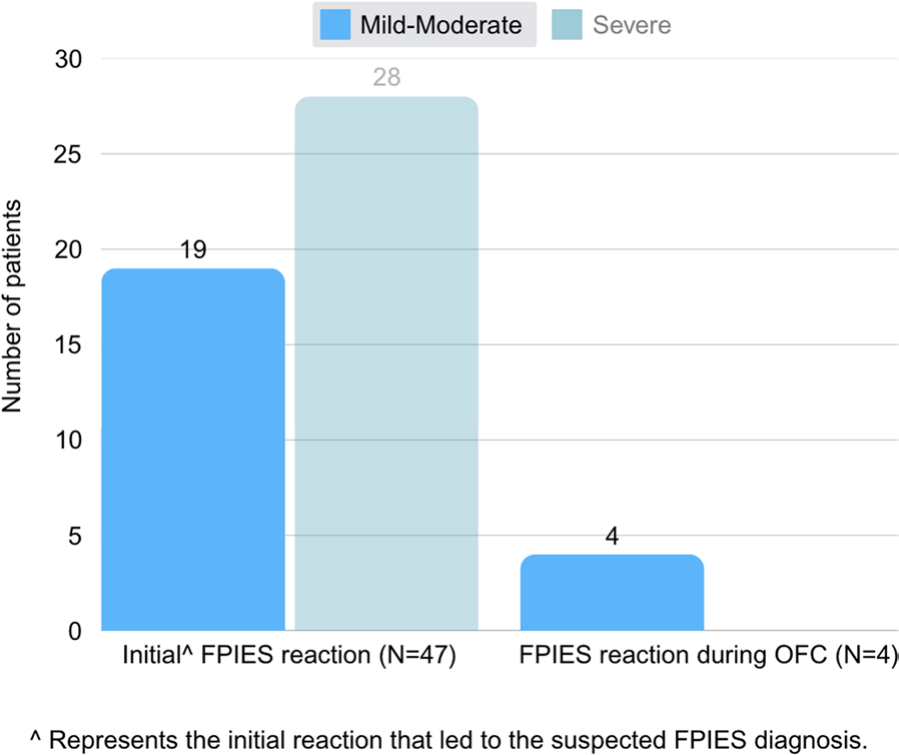
Severity of initial FPIES reactions versus severity of FPIES reactions during OFC

Figure 2 compares the severity of reactions in our study, stratified as either mild-moderate or severe acute FPIES according to Table II in the AAAAI 2017 FPIES guidelines [2]. The initial FPIES reactions highlight that the majority (59.6%) of our study cohort had initial severe acute FPIES reactions that led to their suspected diagnosis. This is compared to our 4 FPIES reactors who paused the protocol, all of which paused due to mild to moderate reactions during the OFC. There were none (0.0%) of severe FPIES reactions during the OFC protocol.

As seen in Table 4, there were no significant associations identified between OFC completion and severity of symptoms (fully adjusted OR 1.05; 95% CI 0.24–4.71; *p* = 0.94); OFC completion and age at onset of symptoms (fully adjusted OR 0.99; 95% CI 0.94–1.02; *p* = 0.58); or OFC completion and age of starting OFC (fully adjusted OR 1.00; 95% CI 0.98–1.02; *p* = 0.90).

**Table 4:** Logistic regression tables of OFC completion

Variable	Severity of symptoms	Age of onset of symptoms	Age of starting OFC
Odds ratio (OR)			
Fully adjusted	1.05	0.99	1.00
Unadjusted	1.13	0.99	1.00
95% confidence interval (CI)			
Fully adjusted	0.24–4.71	0.94–1.02	0.98–1.02
Unadjusted	0.26–4.85	0.97–1.02	0.98–1.01
*p* value			
Fully adjusted	0.94	0.58	0.90
Unadjusted	0.87	0.64	0.85

Compared to foods other than milk/dairy, those who reacted to milk tended to be less likely to complete (fully adjusted OR 0.28; 95% CI 0.07–1.06; *p* = 0.06), which is represented in Table 5.

**Table 5:** Logistic regression analysis of reaction to milk versus other foods

Variable	Reactions milk versus other foods
Odds ratio (OR)	
Fully adjusted	0.28
Unadjusted	0.30
95% confidence interval (CI)	
Fully adjusted	0.07–1.06
Unadjusted	0.08–1.09
*p* value	
Fully adjusted	0.06
Unadjusted	0.07

Long term outcomes were collected based on patient/parent reported symptoms in the 1-year period post full dose escalation. No patients who participated in the novel OFC protocol have contacted the office regarding any recurrence of symptoms, we therefore assume that these patients are symptoms free.

## Discussion

This study presents a retrospective analysis evaluating the efficacy and safety of a novel, gradual OFC protocol tailored specifically for pediatric patients diagnosed FPIES. Through a cautious, incremental approach aimed at minimizing severe reactions, our home OFC protocol demonstrated a high completion rate (80.85%) without significant adverse events. This research highlights potential clinical and practical benefits of a home OFC protocol, emphasizing improved patient safety and tolerability compared to traditional OFC methods.

The ideal OFC protocol should be able to accurately detect changes in patient tolerance to specific food triggers, enabling providers to assess if a patient is progressing safely towards tolerance and responding appropriately to the protocol. Our modified OFC protocol has demonstrated a higher success rate, and a lower incidence of adverse reactions compared to other published protocols. Highlighted in that 80.85% of patients completed the OFC protocol and of those who paused due to reactions, none of the reactions were severe in nature. This suggests that the modified protocol is likely to be successful and safer in FPIES.

Unlike traditional OFC approaches that pose risks of severe reactions, including hypotension and shock, our protocol’s gradual dose escalation over a longer duration enhances patient safety. Additionally, the structured, stepwise dose increases allow for better prediction of long-term tolerance, reducing the need for emergency interventions and supporting a more controlled introduction of trigger foods. Given the higher safety profile and success rates observed, incorporating this modified OFC protocol into both clinical practice and future research could improve approaches to managing patients with FPIES. Among the four patients who paused the protocol due to acute FPIES reactions, only one discontinued due to vomiting, while the remaining three patients stopped due to diarrhea and abdominal pain alone. This distinction is notable, as vomiting is often a hallmark symptom of acute FPIES, yet the majority of patients who reacted did not experience it. This may suggest that a more gradual up-dosing approach could mitigate the more severe emetic responses traditionally seen in acute FPIES reactions.

Additionally, no significant associations were observed between protocol completion and factors such as symptom severity, age at symptom onset, or age at initiation. This implies that these variables did not strongly influence whether patients completed the OFC. Moreover, the data showed a trend where patients who reacted to milk/dairy were less likely to complete the protocol compared to those who reacted to other foods. Although this was not statistically significant, it suggests that milk/dairy may pose a higher risk for protocol non-completion.

Due to the heterogeneity of OFC protocols and inconsistencies in adverse reaction reporting, direct comparison of our safety outcomes to other studies is not feasible. However, examining reported adverse reaction rates in existing OFC protocols provides context for understanding how our findings align with broader trends in FPIES management. Hwang et al. utilized a protocol in which a cumulative dose of 0.15 g protein/kg body weight was administered as a single dose, with resulting severe reactions in 33% of participants (9/27) [18]. Sicherer et al. employed a protocol with cumulative doses administered in increasing amounts every 45–60 min, with mild reactions in 36.3% (4/11) and moderate reactions in 63.6% (7/11) of patients [10]. Similarly, protocols that used equal incremental doses, such as Caubet et al., elicited severe reactions in 19% of patients (14/74) [15]. Examination of multi-day OFC protocols, Infante et al. compared escalating doses (12.5%, 25%, 50%, full dose) administered at 30-min intervals on the same day versus every 48 h. Severe reactions occurred in 39.5% of patients in the same-day cohort compared to 12.5% in the 48-h cohort [19]. Nishimura et al. conducted an OFC protocol over four days, starting with a 1/50th initial dose and increasing once daily if tolerated. Of the eight positive OFCs, six resulted in moderate symptoms, with intravenous fluid resuscitation and/or corticosteroids administered in 50% (4/8) of the patients [20]. The modification of the current OFC protocol, based on international consensus guidelines, might be required for patients with severe acute FPIES. Our modified protocol, which begins with a first loading dose of 1/100, elicited fewer adverse reactions in our population.

Our study has several strengths beyond the emphasis on patient safety, the data collection and analysis examine a wide range of demographic and clinical variables that may impact protocol success. By exploring factors such as age, allergic comorbidities, and specific food triggers, the study offers insights for clinical application. The study also differentiates between patients who successfully completed the protocol and those who did not, providing insights into the specific challenges faced by non-completers. This differentiation allows for targeted refinement of the protocol to address these challenges and better tailor it to patient needs. Targeting specifics into why patients would not complete the protocol could be an avenue for future research.

One limitation of the modified OFC protocol is its extended duration, gradually increasing doses every 2 to 4 weeks over 10 months. This slow progression could intersect with the natural outgrowth of FPIES in children. Therefore, it may be challenging to distinguish whether the observed tolerance is due to the safety of the protocol or the natural resolution of FPIES. This limitation highlights the need for further research to differentiate between these two factors. Another limitation is the absence of a standardized protein measurement for the food triggers used in the protocol. Without precise quantification of the protein dose, it is difficult to ensure consistency and reproducibility of the challenge doses across different patients. This variability could impact the interpretation of the results and the overall efficacy of the protocol. Future studies should consider implementing standardized protein measurements to enhance the reliability of OFC protocols. We note that protein may not be the cause of FPIES. Additionally, although the primary aim of the protocol is to confirm tolerance to suspected food triggers safely, there is a possibility that the gradual increase in doses could contribute to desensitization. If the protocol inadvertently desensitizes patients, it may mask the true severity of FPIES reactions, leading to overestimation of the safety profile of the protocol. Additionally, although the primary aim of the protocol is to confirm tolerance to suspected food triggers safely, there is a possibility that the gradual increase in doses could contribute to some form of desensitization. If this type of protocol desensitizes patients, it may become a treatment for FPIES. The retrospective nature of our cohort study yields limitations, prospective studies with larger, diverse populations are needed to validate the findings and ensure their applicability to different clinical settings. Furthermore, long-term outcomes were collected based on instructions given to patients/parents to contact the treating physician with any recurrence of symptoms during the 1-year period post full dose escalation. No formal follow up may have led to inconsistent reporting of symptom free periods. Lastly, our strict inclusion criteria led to 10% of the study cohort excluded from analysis due to non-FPIES reactions, which may introduce biases to our study sample.

Overall, our study highlights the potential benefits of a modified OFC protocol in safely managing FPIES, but it also underscores the need for further research to address the identified limitations. By refining these protocols and conducting more comprehensive studies, we can improve the care and outcomes for patients with FPIES. This study contributes to the growing body of knowledge on FPIES and provides a foundation for future research aimed at optimizing OFC protocols and enhancing patient safety.

## Conclusion

The decision to implement an OFC protocol in managing FPIES is complex, necessitating a nuanced evaluation of risks and benefits. While current protocols for OFCs are established, concerns arise regarding the risk of severe reactions such as hypotension and shock. Our retrospective cohort study assessed the efficacy of a modified home-based, slower OFC protocol in mitigating these risks. We found our slower gradual approach was highly tolerable, with 80.85% of patients completing the OFC protocol without significant reactions. This study’s findings will suggest a potential need to refine the approach to FPIES OFC’s and management.

## Data Availability

The data that support the findings of this study are not openly available due to reasons of patient privacy and are not available for request due to similar reasoning. Data is located in controlled access data storage by the corresponding author.

## Abbreviations

AAAAI: American Academy of Allergy, Asthma & Immunology
FPIAP: Food protein–induced allergic proctocolitis
FPIES: Food protein–induced enterocolitis syndrome
IgE: Immunoglobulin E
OFC: Oral food challenge
OR: Odds ratio
95% CI: 95% confidence intervals
